# Is HER2-Low Breast Cancer a Distinct Prognostic Entity? Real-World Survival Outcomes from a Cohort in Colombia

**DOI:** 10.3390/cancers18203322

**Published:** 2026-10-15

**Authors:** Edgar Fabián Manrique-Hernández, Maricel Licht-Ardila, Johan Danilo Torres Rosas, Jorge Andrés Rincón Pallares, Michael Steven Villabona Díaz, Lady Katerine Aldana Galvis, Kendrys Hoyos, Alexandra Hurtado-Ortiz, Carlos Calderón Cortés, Diego Gómez-Abreo

**Affiliations:** 1 Epidemiology Department, Hospital Internacional de Colombia (HIC), Fundación Cardiovascular de Colombia (FCV), Fundación Universitaria FCV, Bucaramanga 680001, Colombia; mlichtar@gmail.com (M.L.-A.); alexandrajhop@gmail.com (A.H.-O.); 2 Oncology Department, Hospital Internacional de Colombia (HIC), Fundación Cardiovascular de Colombia (FCV), Fundación Universitaria FCV, Bucaramanga 680001, Colombia; torresdaniloj@hotmail.com (J.D.T.R.); jorgerinconpallares@gmail.com (J.A.R.P.); michaelvillabona08@gmail.com (M.S.V.D.); ladyaldana0531@gmail.com (L.K.A.G.); kendryshoyos@fcv.org (K.H.); ccalderonha@gmail.com (C.C.C.); diegogomezabreo@gmail.com (D.G.-A.)

**Keywords:** breast neoplasms, HER2 protein, survival analysis, disease-free survival, prognosis, cohort studies, neoplasm staging

## Abstract

**Background/Objectives:** The HER2-low classification has redefined therapeutic strategies, but its prognostic significance remains uncertain. We compared overall survival (OS) and progression-free survival (PFS) across HER2 categories (HER2-low, HER2-negative, HER2-positive) in a real-world cohort. **Methods:** This was a single-center retrospective cohort study conducted at a high-complexity cancer center in Colombia; it included 373 patients with invasive breast cancer classified as HER2-positive (IHC 3+ or ISH+), HER2-low (IHC 1+ or 2+/ISH−), or HER2-negative (IHC 0). Survival was estimated by Kaplan–Meier. Multivariable Cox models were adjusted for age, clinical stage, histological grade, and hormone receptor status, and PFS was additionally analyzed separately in stage I–III and stage IV disease. **Results:** The cohort included HER2-low (38.3%), HER2-negative (39.7%), and HER2-positive (22.0%) tumors. HER2-low tumors showed lower grade and higher hormone receptor expression (*p* < 0.001), without better survival. Three-year OS (81.8%, 76.5%, 62.8%; *p* = 0.178) and PFS (*p* = 0.227) did not differ. Advanced stage remained the main determinant of mortality, whereas the association with high grade was attenuated. HER2-low was not independently associated with OS (HR 0.67; 95% CI 0.31–1.45) or PFS (HR 0.77; 95% CI 0.40–1.49). Hormone receptor positivity was associated with lower mortality (HR 0.43; 95% CI 0.21–0.88). PFS did not differ across HER2 categories in stage I–III (*p* = 0.307) or stage IV disease (*p* = 0.068). **Conclusions:** HER2-low status was not independently associated with survival. Its more favorable clinicopathological profile was largely explained by higher hormone receptor expression, which was itself associated with survival once included in the models.

## Simple Summary

Breast cancer is commonly classified according to the presence or absence of a protein called HER2, which helps guide treatment decisions. Recently, a new category known as HER2-low has gained attention because patients with these tumors may benefit from newly developed targeted therapies. However, it remains unclear whether HER2-low tumors have a different prognosis from other breast cancer types. We analyzed real-world data from 373 patients treated at a specialized cancer center in Colombia to compare survival outcomes among patients with HER2-low, HER2-positive, and HER2-negative breast cancers. Although HER2-low tumors showed more favorable biological characteristics, patients did not experience better overall or progression-free survival after accounting for clinical stage, histological grade, and hormone receptor status. These findings suggest that HER2-low should not be considered a distinct prognostic category but remains clinically important because it identifies patients who may benefit from emerging targeted treatments.

## 1. Introduction

Breast cancer (BC) remains the most frequently diagnosed malignancy and a leading cause of cancer-related mortality among women worldwide, with a steadily increasing incidence and substantial heterogeneity in clinical outcomes [1,2]. Molecular classification guides therapeutic decision-making, particularly through the assessment of hormone receptor status and human epidermal growth factor receptor 2 (HER2) expression [3].

Historically, HER2-positive BC, defined by HER2 overexpression or gene amplification, has been associated with aggressive tumor biology; however, outcomes have improved markedly with effective HER2-directed therapies [4]. In contrast, HER2-negative disease comprises tumors without HER2 overexpression or amplification and includes both IHC 0 tumors and HER2-low expression categories. This group has traditionally lacked specific HER2-directed treatment options.

More recently, attention has shifted toward a subgroup within HER2-negative disease characterized by low levels of HER2 expression, referred to as HER2-low [5,6]. Typically defined as tumors with immunohistochemistry (IHC) scores of 1+ or 2+ without gene amplification, HER2-low is increasingly understood as part of a biological continuum rather than a distinct molecular subtype [3]. Recent international expert consensus statements have similarly emphasized that HER2-low should currently be interpreted as a therapeutically actionable expression category rather than as an independent molecular subtype, while highlighting the need for standardized pathological assessment [7,8]. This concept is particularly relevant given the interplay between HER2 signaling and hormone receptor pathways, as well as the recognized intratumoral heterogeneity in HER2 expression.

The clinical relevance of HER2-low classification has been reinforced by the development of antibody–drug conjugates (ADCs), such as trastuzumab deruxtecan, which have demonstrated significant survival benefits in patients with HER2-low metastatic disease. These findings challenge the traditional binary classification of HER2 status and position HER2-low as a therapeutically actionable category [9].

Despite these advances, the prognostic significance of HER2-low expression remains unclear. While some studies have reported improved survival compared with HER2-negative tumors, particularly in hormone receptor–positive disease, others have not observed significant differences after adjustment for key clinicopathological variables [6,10,11]. These inconsistencies may be explained by underlying biological confounding, including the higher prevalence of hormone receptor positivity within the HER2-low subgroup. Importantly, prognostic significance and therapeutic actionability are distinct concepts. A biomarker may identify patients who could benefit from a specific therapy without being independently associated with the natural course of the disease or survival outcomes. Accordingly, the present study focused specifically on the prognostic association between HER2 expression categories and survival outcomes and was not designed to evaluate the predictive value of HER2-low status for response to HER2-directed therapies.

Most evidence on the prognostic value of HER2-low expression comes from North American, European, and East Asian cohorts. Data from Latin America are scarce. A recent systematic review of Hispanic and Latin American women identified prevalence estimates only, with no analyses of survival outcomes [12]. It is therefore unknown whether the prognostic patterns reported elsewhere apply to populations that differ in stage at presentation, treatment availability, and access to HER2-directed therapy. Therefore, we aimed to compare overall survival (OS) and progression-free survival (PFS) across HER2 expression categories (HER2-low, HER2-negative, and HER2-positive) in patients with breast cancer treated at a high-complexity cancer center in Colombia.

## 2. Materials and Methods

We conducted a single-center, retrospective real-world cohort study using real-world data from adult patients diagnosed with breast cancer (BC) and treated at the Breast Cancer Center of Excellence of a high-complexity referral hospital in northeastern Colombia. The center provides multidisciplinary care involving medical oncology, breast surgery, pathology, radiology, radiation oncology, nuclear medicine, plastic surgery, psychology, nursing, social work, nutrition, pain management, and palliative care.

**Figure 1:**
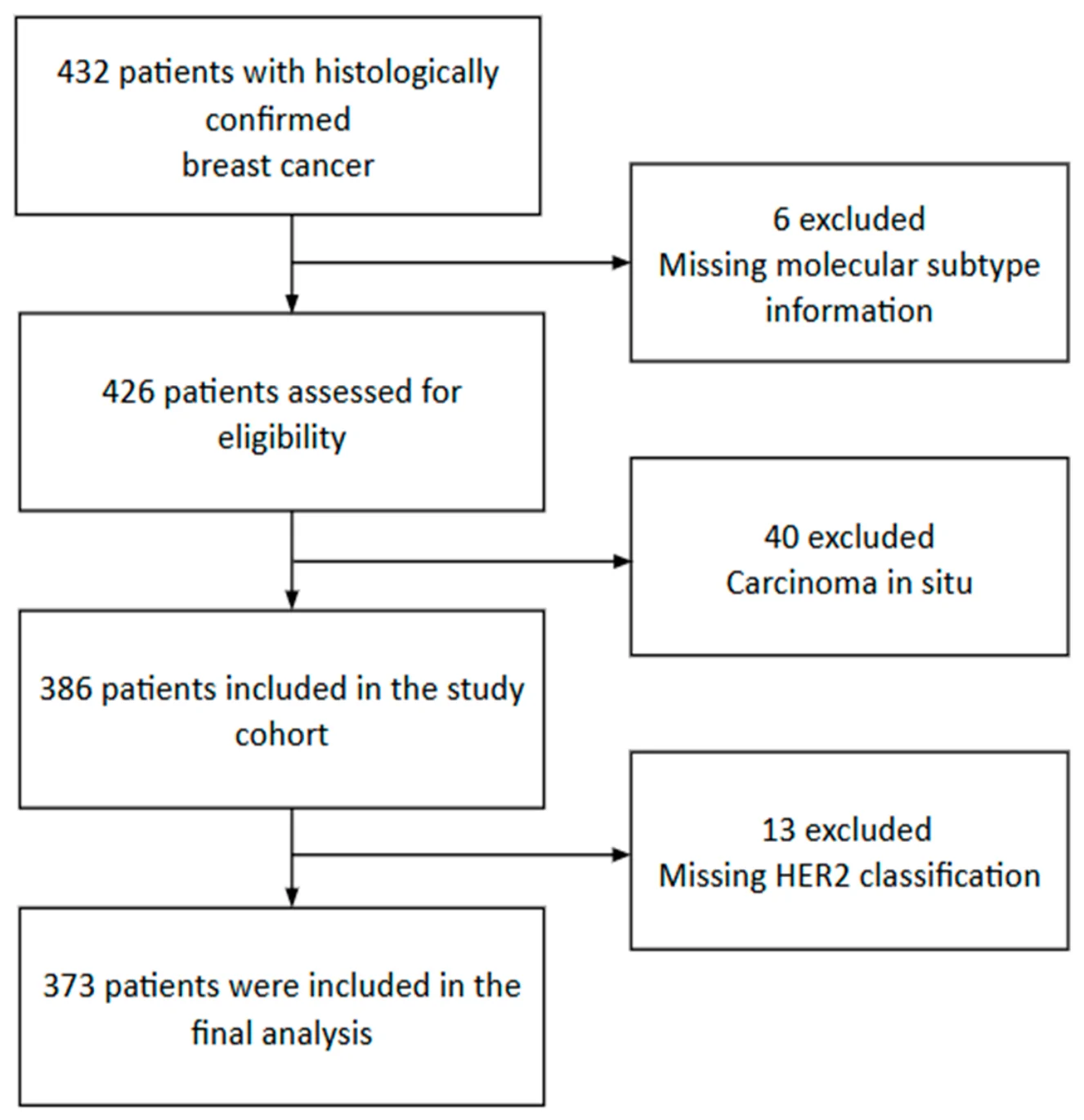
Flowchart of patient selection according to HER2 classification. A total of 432 patients with histologically confirmed breast cancer were initially identified; six were excluded because of missing molecular subtype information. Among the 426 patients assessed for eligibility, 40 with carcinoma in situ were excluded. Of the remaining 386 patients, 13 were excluded because of missing HER2 classification, resulting in a final analytic sample of 373 patients.

The study period extended from 1 June 2022 to 30 November 2025. Patients aged ≥18 years with histologically confirmed BC were eligible for inclusion. Exclusion criteria were refusal of oncologic treatment at the institution, carcinoma in situ, and missing HER2 data required for classification by immunohistochemistry (IHC) or in situ hybridization (ISH). Patient selection is shown in Figure 1.

HER2 expression was classified by IHC and ISH according to current clinical guidelines [13]. HER2-positive tumors were defined as IHC 3+ or IHC 2+ with ISH amplification, HER2-low as IHC 1+ or IHC 2+ with negative ISH, and HER2-negative as IHC 0. HER2-negative was used as the comparator/reference category throughout the analyses. For consistency throughout the manuscript, tumors with IHC 0 were referred to as HER2-negative rather than HER2-zero. HER2 testing was performed as part of routine clinical pathology practice at the institution. For this retrospective study, HER2 status was extracted from the original pathology reports, and no centralized pathological reassessment was performed.

Sociodemographic variables included sex, age at diagnosis, and health insurance status. Clinical variables comprised clinical stage, histological grade, hormone receptor status (estrogen and progesterone receptors), Ki-67 proliferation index, treatment modality, response to first-line therapy, distant metastatic status (presence, number, and sites), disease progression, and survival outcomes. For descriptive purposes, first-line treatment modalities were grouped according to the principal therapeutic strategy documented in the medical record. The timing of distant metastatic disease was determined from the clinical records according to the date on which metastatic involvement was documented. Tumor staging was determined according to the TNM classification (8th edition) based on pathological findings and imaging studies, including computed tomography and bone scintigraphy when clinically indicated. Hormone receptor status was classified as positive when estrogen and/or progesterone receptor expression was documented in ≥1% of tumor cells, and as negative when both receptors were expressed in <1% of tumor cells.

Time-to-event outcomes were calculated from the date of diagnosis. OS was defined as the time from diagnosis to death from any cause. PFS was defined as the time from diagnosis to documented disease progression or death, whichever occurred first. In patients with early-stage disease treated with curative intent, progression included documented locoregional or distant recurrence, whereas in patients with advanced disease it reflected clinical or radiological disease progression. Because these two settings correspond to clinically different endpoints, PFS was analyzed both in the whole cohort and separately in patients with stage I–III disease treated with curative intent and in patients with stage IV disease. The time origin was the date of diagnosis in both strata, so that the stage I–III analysis approximates recurrence-free survival and the stage IV analysis reflects progression of established metastatic disease. Disease progression was defined as radiological or clinical evidence of tumor progression or the development of new metastatic lesions. Patients without an event were censored at the date of their last documented follow-up. For the assessment of follow-up completeness, patients without a recorded death and with no documented clinical or telephone contact for more than 12 months before the administrative data cutoff were operationally classified as lost to follow-up. This classification was used to describe follow-up completeness and did not modify time-to-event censoring, which remained based on the date of the last known contact.

As part of routine clinical practice, patients underwent longitudinal surveillance through scheduled ambulatory consultations and standardized telephone assessments. For the present retrospective analysis, demographic, clinical, and laboratory information was extracted from institutional electronic health records and complementary clinical databases. Trained members of the research team entered the collected information into REDCap (Research Electronic Data Capture), version 13.4.13 (Vanderbilt University, Nashville, TN, USA), under project number 563 [14]. Data accuracy and completeness were assessed by duplicate verification and systematic comparison of the database entries with the original source records.

Categorical data were described using absolute and relative frequencies, whereas continuous variables were summarized as medians and interquartile ranges (IQRs). Differences among HER2 expression groups were evaluated using the chi-square test or Fisher’s exact test, as appropriate, for categorical variables, and the Kruskal–Wallis test for continuous variables. Time-to-event distributions were estimated using the Kaplan–Meier method, and survival curves were compared using the log-rank test. Median follow-up was estimated using the reverse Kaplan–Meier method, with follow-up calculated from the date of diagnosis to the date of death or last documented clinical contact.

Multivariable Cox proportional hazards regression models were constructed to assess the independent association between HER2 classification and survival outcomes (OS and PFS). Models were adjusted a priori for age (continuous), clinical stage (III–IV vs. I–II), histological grade (grade 3 vs. 1–2), and hormone receptor status (positive vs. negative). Hormone receptor status was retained in the principal models because it is the main biological confounder of the comparison between HER2 expression categories. Ki-67 and first-line treatment were not included, since adding them would have increased the number of estimated parameters beyond what the available number of events could support, and residual confounding related to proliferative activity and treatment selection therefore cannot be excluded. Because only 32 patients had stage IV disease, stage-specific multivariable models were not fitted, and the separate analyses of PFS by disease setting were restricted to Kaplan–Meier estimation and the log-rank test. Results are reported as hazard ratios (HR) with 95% confidence intervals (95% CI). Given the limited number of outcome events, a post hoc minimum detectable effect calculation was performed for the primary comparison between HER2-low and HER2-negative tumors using Schoenfeld’s approximation, assuming a two-sided α of 0.05 and 80% power.

Missing data were not imputed. Descriptive and bivariate analyses were conducted using the available observations for each variable, and denominators therefore varied according to data availability. Multivariable Cox regression analyses were performed using complete-case analysis for the covariates included in each model.

Overall model performance was examined using the likelihood-ratio test, while compliance with the proportional hazards assumption was verified through the analysis of Schoenfeld residuals. Statistical significance was defined using a two-tailed threshold of *p* < 0.05. All statistical procedures were conducted in Stata version 16 (StataCorp LLC, College Station, TX, USA).

The research was performed in compliance with applicable national regulations and international ethical principles. Under Resolution 8430 of 1993 issued by the Colombian Ministry of Health, the study was categorized as minimal-risk research. Confidentiality was protected by anonymizing the study records and limiting database access to authorized personnel. The protocol received approval from the Institutional Ethics Committee of Fundación Cardiovascular de Colombia (approval code CEI-2024-07461-42).

## 3. Results

A total of 373 patients were included, with a balanced distribution across HER2 groups: HER2-low (38.3%), HER2-negative (39.7%), and HER2-positive (22.0%). Age and clinical stage were comparable between groups (*p* = 0.110 and *p* = 0.225), with stage II being the most frequent. High-grade tumors (grade 3) were more frequent in HER2-negative (40.1%) and HER2-positive (42.7%) groups, whereas HER2-low tumors were predominantly grade 2 (69.0%) (*p* < 0.001) (Table 1).

Consistently, HER2-low tumors showed higher hormone receptor expression, while HER2-positive tumors exhibited lower ER and PR levels (*p* < 0.001). Overall, 293 patients (78.6%) had hormone receptor–positive tumors. Hormone receptor positivity was more frequent among HER2-low tumors (124/143, 86.7%) than among HER2-negative (116/148, 78.4%) and HER2-positive tumors (53/82, 64.6%) (*p* = 0.001). Ki-67 was higher in HER2-positive tumors and lower in HER2-low tumors (*p* < 0.001). Complete response rates were 42.7% in HER2-positive, 33.8% in HER2-negative, and 30.8% in HER2-low tumors, with no significant difference between groups (*p* = 0.076). Distant metastatic disease occurred in 13.9% of patients, without differences across HER2 categories (*p* = 0.388) (Table 1).

Response to first-line treatment differed significantly according to clinical stage (*p* < 0.001). Complete response was more frequent among patients with stage I disease (35/60, 58.3%) and decreased across stage II (61/152, 40.1%), stage III (31/129, 24.0%), and stage IV disease (2/32, 6.3%). Partial response was most frequent in stage III disease (72/129, 55.8%), compared with stage II (65/152, 42.8%), stage I (21/60, 35.0%), and stage IV disease (7/32, 21.9%). Stable disease and progressive disease were uncommon in stages I–III but were each observed in 8 of 32 patients (25.0%) with stage IV disease. Treatment response was not evaluated in 52 patients overall (13.9%).

Among patients with metastatic disease (*n* = 52), bone (51.9%) and lung (46.2%) were the most frequent sites, with no significant differences between groups. Central nervous system (CNS) involvement was the only site that differed significantly according to HER2 classification (*p* = 0.011), being more frequent in HER2-positive tumors (55.6%) than in HER2-negative (20.0%) and HER2-low tumors (5.6%). Other metastatic sites were less frequent and similarly distributed (Table 2).

First-line treatment strategies differed significantly across HER2 groups (*p* < 0.001). HER2-positive tumors were predominantly treated with anti-HER2–based regimens (62.2%). In contrast, HER2-negative tumors more frequently received dose-dense anthracycline-based chemotherapy (27.0%) and surgical management (27.0%). HER2-low tumors exhibited a more heterogeneous treatment pattern, including dose-dense anthracyclines (23.8%), surgery (14.7%), and other systemic therapies (25.2%) (Table 3).

**Figure 2:**
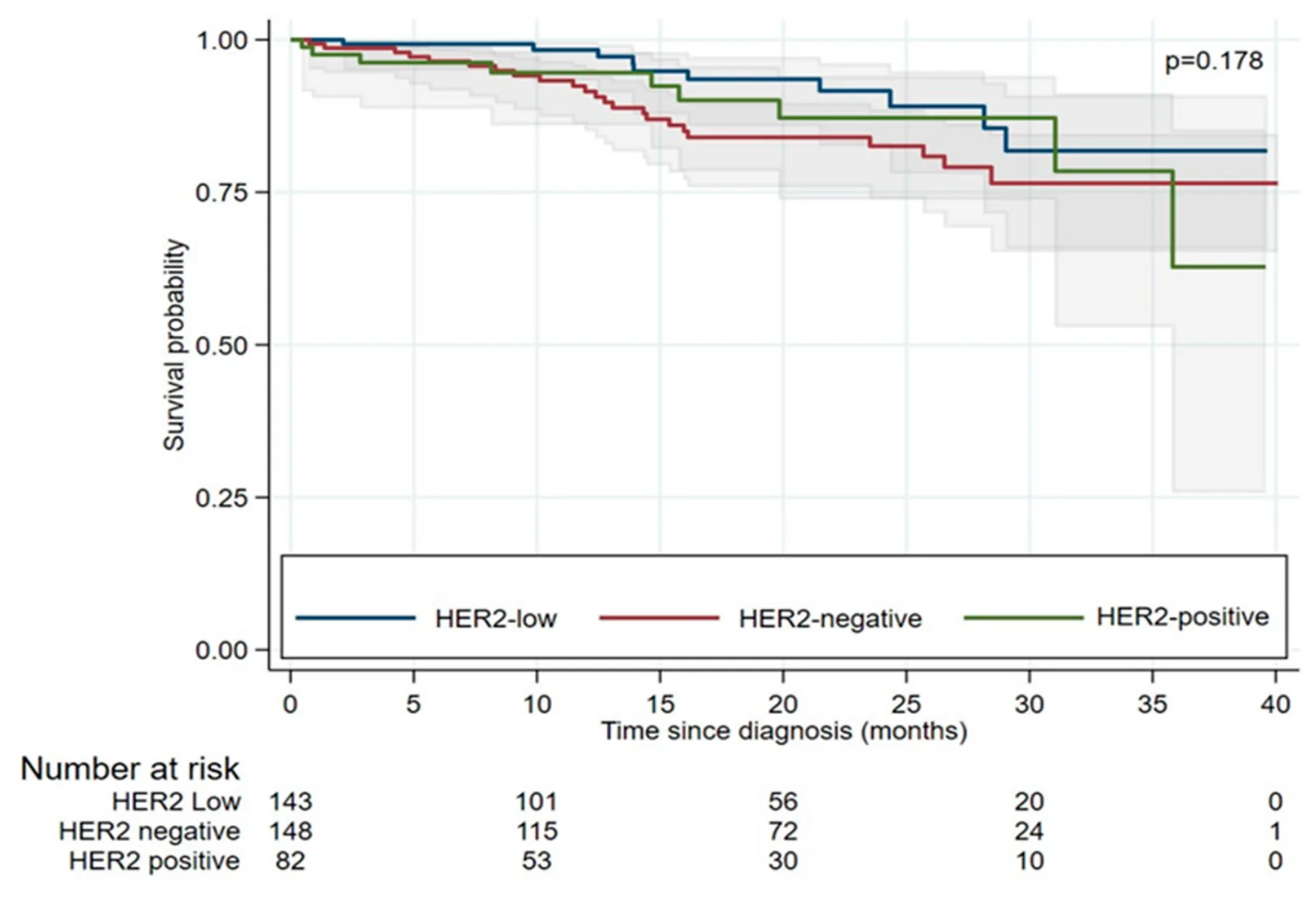
Overall Survival According to HER2 Classification. Overall survival (OS) was estimated using the Kaplan–Meier method according to HER2 classification (HER2-low, HER2-negative, and HER2-positive). The grey shaded areas represent the 95% confidence intervals (95% CIs). Differences between groups were assessed using the log-rank test.

The median follow-up was 19.3 months (95% CI, 17.5–21.0), with a maximum observed follow-up of 40.1 months. Overall, 42 of 373 patients (11.3%) met the prespecified operational definition of loss to follow-up, defined as having no documented clinical or telephone contact for more than 12 months before the administrative cutoff. During follow-up, 42 deaths were recorded among the 373 patients. The estimated 1-year OS was 98.3% (95% CI, 93.4–99.6) for HER2-low tumors, 91.5% (95% CI, 85.2–95.2) for HER2-negative tumors, and 94.6% (95% CI, 86.1–97.9) for HER2-positive tumors. At 3 years, OS was 81.8% (95% CI, 65.8–90.8), 76.5% (95% CI, 65.3–84.5), and 62.8% (95% CI, 25.9–85.2), respectively. Although the HER2-positive group showed descriptively lower OS at 3 years, no statistically significant differences were observed across HER2 categories (log-rank test: χ^2^(2) = 3.46, *p* = 0.178) (Figure 2).

**Figure 3:**
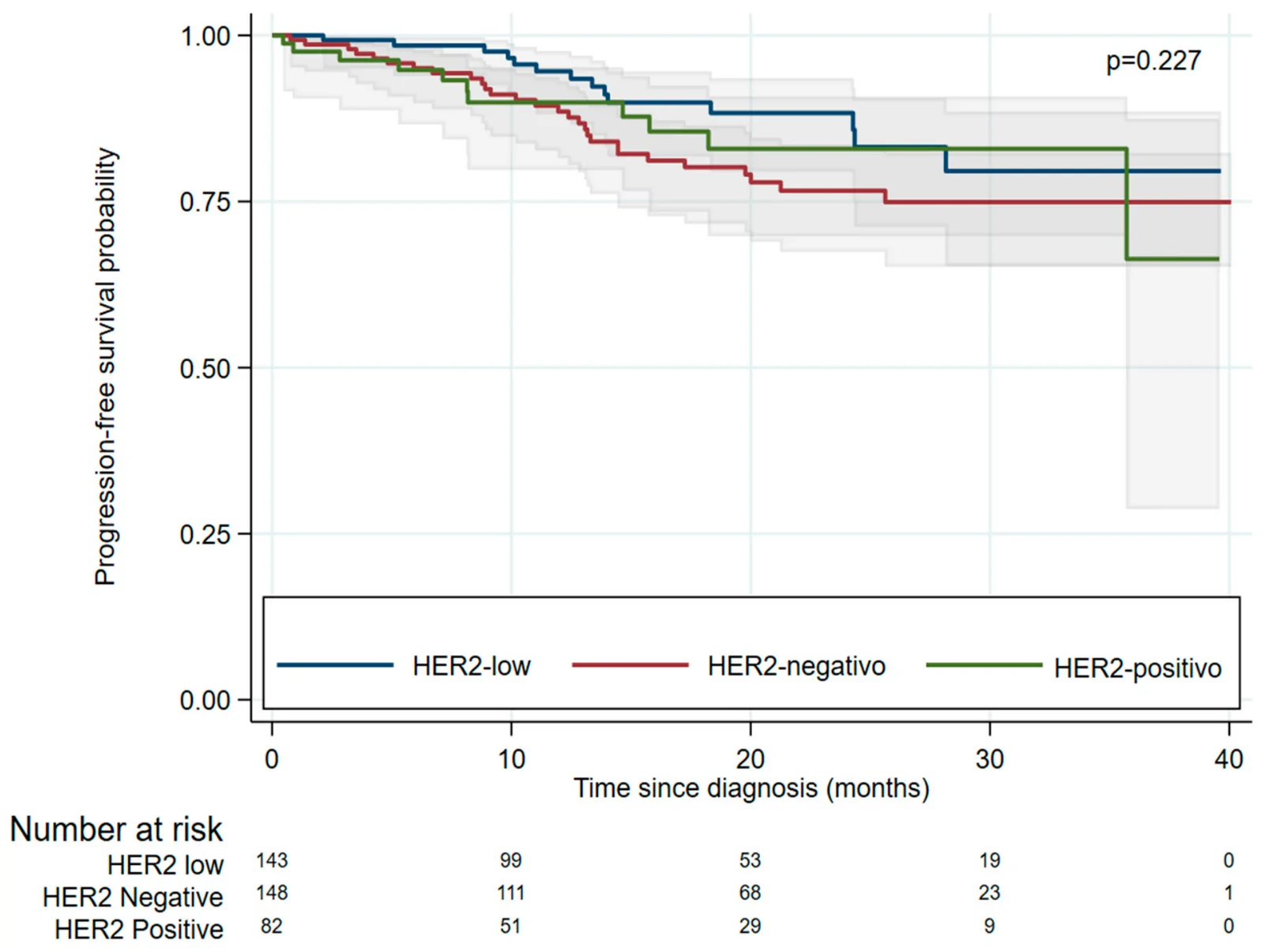
Progression-Free Survival According to HER2 Classification. Progression-free survival (PFS) estimated using the Kaplan–Meier method according to HER2 classification (HER2-low, HER2-negative, and HER2-positive). The grey shaded areas represent the 95% confidence intervals (95% CIs). Differences between groups were assessed using the log-rank test.

Overall, the Kaplan–Meier curves for PFS demonstrated substantial overlap across HER2 classification groups, indicating comparable PFS probabilities over time. During a follow-up of up to 40 months, 53 PFS events, defined as disease progression or death from any cause, whichever occurred first, were observed among 373 patients. At the end of the observed follow-up, the estimated PFS was 79.6% (95% CI: 65.4–88.5) for the HER2-low group, 74.9% (95% CI: 65.3–82.2) for the HER2-negative group, and 66.4% (95% CI: 28.9–87.3) for the HER2-positive group. Although the HER2-positive group showed a descriptively lower PFS probability, differences across HER2 classification groups were not statistically significant (log-rank test: χ^2^(2) = 2.96, *p* = 0.227) (Figure 3).

**Figure 4:**
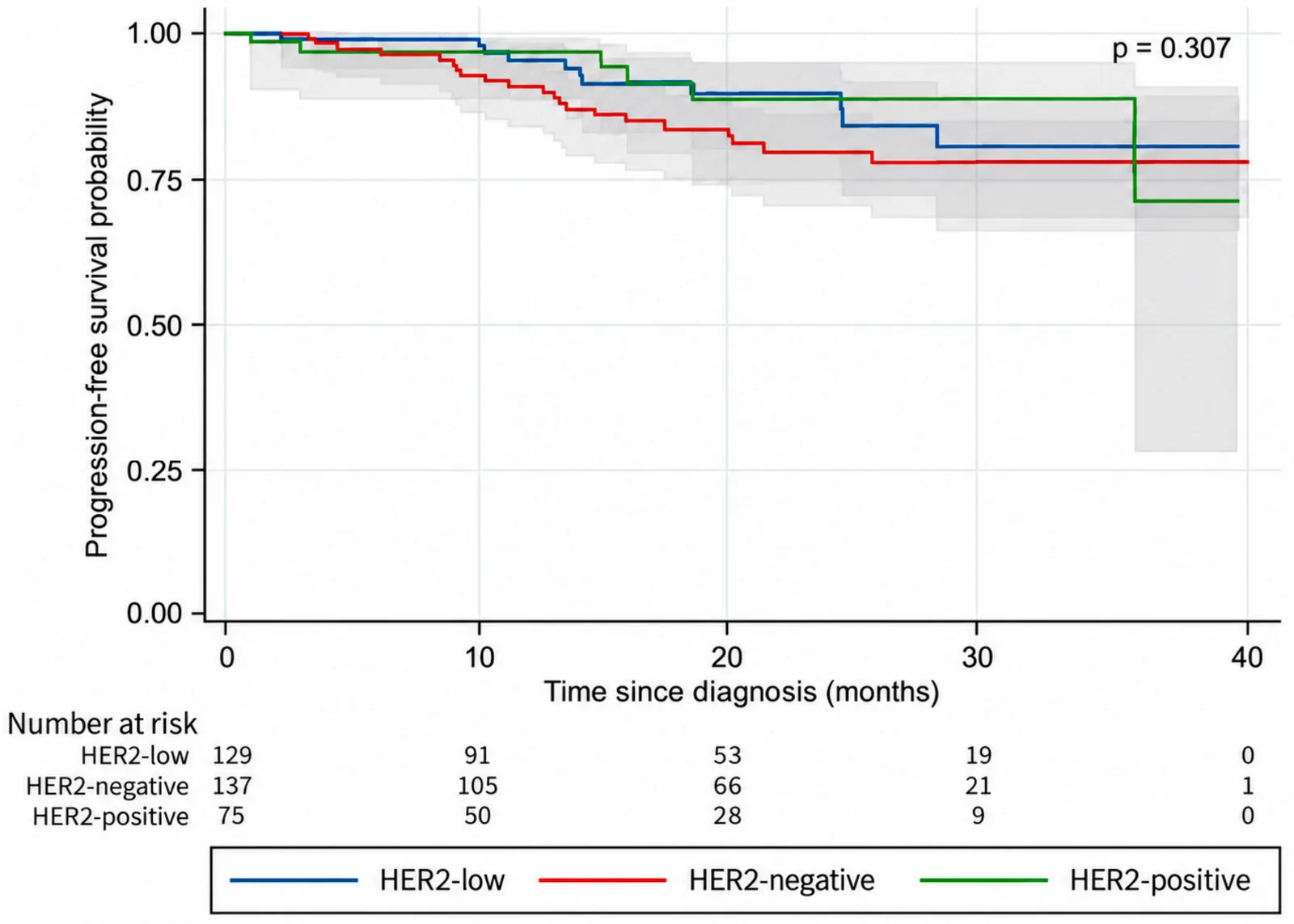
Progression-Free Survival According to HER2 Classification in Patients with Stage I–III Disease. Progression-free survival estimated using the Kaplan–Meier method in the 341 patients with stage I–III disease, with 95% confidence intervals shaded and numbers at risk shown below the curves. Differences between groups were assessed using the log-rank test.

Because the pooled PFS analysis combined patients treated with curative intent and patients with advanced disease, PFS was additionally examined separately by disease setting. Among the 341 patients with stage I–III disease, 38 events were recorded (HER2-negative 21/137, HER2-low 11/129, HER2-positive 6/75). At the end of the observed follow-up, PFS was 81.4% (95% CI, 66.6–90.1) for HER2-low, 78.8% (95% CI, 68.8–85.9) for HER2-negative, and 71.6% (95% CI, 27.7–91.7) for HER2-positive tumors, with no significant difference across groups (log-rank test: χ^2^(2) = 2.36, *p* = 0.307) (Figure 4).

**Figure 5:**
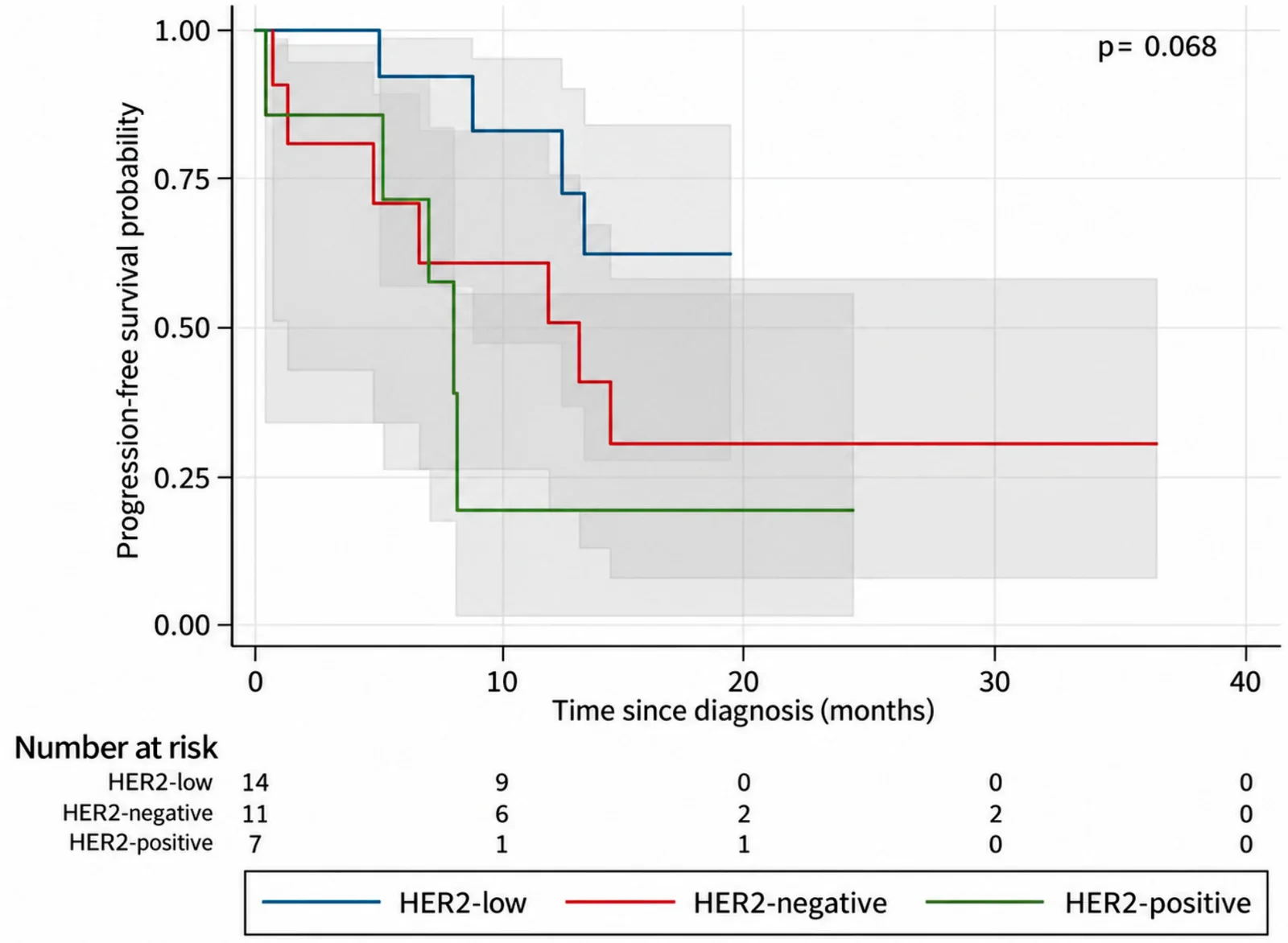
Progression-Free Survival According to HER2 Classification in Patients with Stage IV Disease. Exploratory Kaplan–Meier analysis in the 32 patients with stage IV disease, with 95% confidence intervals shaded and numbers at risk shown below the curves. Differences between groups were assessed using the log-rank test. Estimates are imprecise because of the small number of patients and events.

Among the 32 patients with stage IV disease, 16 events were recorded (HER2-negative 7/11, HER2-low 4/14, HER2-positive 5/7). PFS estimates were 62.3% (95% CI, 27.4–84.1) for HER2-low, 30.3% (95% CI, 7.2–58.2) for HER2-negative, and 19.1% (95% CI, 0.9–55.6) for HER2-positive tumors. The difference across HER2 categories did not reach statistical significance (log-rank test: χ^2^(2) = 5.39, *p* = 0.068), and given the small number of patients and the width of the confidence intervals, this comparison should be regarded as exploratory (Figure 5).

The multivariable OS model included 368 patients with 41 deaths, and the PFS model included 368 patients with 53 events. In multivariable Cox regression models adjusted for age, clinical stage, histological grade, and hormone receptor status, HER2 classification was not independently associated with OS or PFS (global test for HER2: *p* = 0.461 for OS and *p* = 0.598 for PFS). Advanced clinical stage remained the strongest predictor of mortality (OS: HR 6.09; 95% CI 2.64–14.02; *p* < 0.001) and of progression or death (PFS: HR 4.24; 95% CI 2.21–8.10; *p* < 0.001). Hormone receptor–positive disease was associated with lower mortality (OS: HR 0.43; 95% CI 0.21–0.88; *p* = 0.020), with a similar direction for PFS that did not reach significance (HR 0.57; 95% CI 0.30–1.06; *p* = 0.078). After hormone receptor status was included, high histological grade was no longer independently associated with outcome (OS: HR 2.04; 95% CI 0.96–4.35; *p* = 0.065; PFS: HR 1.71; 95% CI 0.90–3.28; *p* = 0.102). HER2-low status was not associated with OS (HR 0.67; 95% CI 0.31–1.45; *p* = 0.312) or PFS (HR 0.77; 95% CI 0.40–1.49; *p* = 0.443) (Table 4). Both models were globally significant (OS: likelihood-ratio χ^2^(6) = 55.34; PFS: χ^2^(6) = 42.51; both *p* < 0.001). The proportional hazards assumption was not violated for either OS (global Schoenfeld test: χ^2^(6) = 6.88, *p* = 0.332) or PFS (χ^2^(6) = 5.38, *p* = 0.496), supporting the validity of the models. For the comparison between HER2-low and HER2-negative tumors, the minimum detectable hazard ratios were 2.65 for OS and 2.37 for PFS, with equivalent protective hazard ratios of 0.38 and 0.42, respectively.

## 4. Discussion

This real-world cohort study evaluated the prognostic relevance of HER2-low breast cancer compared with HER2-negative and HER2-positive disease. Despite differences in clinicopathological characteristics and treatment patterns across HER2 categories, HER2-low status was not significantly associated with OS or PFS after adjustment for age, clinical stage, histological grade, and hormone receptor status. These findings suggest that HER2-low expression provided limited additional prognostic information in this predominantly non-metastatic cohort. To our knowledge, this is one of the first cohorts from Latin America to report OS and PFS by HER2 expression category. The existing regional literature has documented the prevalence of HER2-low tumors without linking it to clinical outcomes [12].

A notable finding was the discrepancy between the more favorable clinicopathological profile of HER2-low tumors and the absence of a clear survival advantage. HER2-low tumors were characterized by lower histological grade, higher hormone receptor expression, and lower proliferative activity, consistent with previous reports describing greater enrichment of luminal features in this group [3,6,15]. This pattern suggests that the apparently more indolent phenotype of HER2-low tumors may be partly related to hormone receptor biology and treatment selection rather than to low HER2 expression itself. When hormone receptor status was added to the multivariable models, it was itself associated with lower mortality, while HER2-low status remained unrelated to survival. This pattern is consistent with the interpretation that the apparently indolent behavior of HER2-low tumors reflects hormone receptor biology rather than low HER2 expression as such.

Our findings are consistent with previous studies that have questioned the independent prognostic relevance of HER2-low expression. Tarantino et al. analyzed 5235 patients with stage I–III ERBB2-negative breast cancer and reported that ERBB2-low expression had no independent prognostic significance after accounting for hormone receptor status [5]. Similarly, Schettini et al. analyzed clinicopathological and molecular data from 3689 patients with HER2-negative breast cancer and conducted an exploratory OS analysis in 1304 patients with advanced disease. No significant differences in OS were observed between HER2-low and HER2-0 tumors, including analyses stratified by hormone receptor status [3]. These findings, together with the biological heterogeneity observed across HER2-expressing breast cancers, argue against interpreting HER2-low expression alone as a clearly defined prognostic entity [16].

Published cohorts have generally examined HER2-low expression either in early-stage or in advanced disease. The Tarantino cohort excluded stage IV disease and consisted mostly of stage I tumors [5], whereas the survival analyses by Schettini et al. and Holthuis et al. were restricted to advanced and metastatic disease [3,17]. Our cohort spans both settings: 16.1% of patients presented with stage I disease, 43.2% with stage III or IV, and patients with metastatic disease at diagnosis were included. The absence of an independent association between HER2-low status and survival was therefore observed across a broader range of clinical stages than in most individual series. To address the clinical heterogeneity of a pooled endpoint, PFS was also examined separately in patients treated with curative intent and in patients with stage IV disease. No differences across HER2 categories emerged in stage I–III disease, and the stage IV comparison, although suggesting more favorable outcomes among HER2-low tumors, rests on 32 patients and 16 events and cannot support inference. Separating the two settings did not change the conclusion drawn from the pooled analysis, but it makes explicit that the endpoint captures recurrence after curative treatment in one group and progression of established metastatic disease in the other.

Nevertheless, the available prognostic evidence remains heterogeneous. In a population-based cohort of patients with metastatic breast cancer, Holthuis et al. found no marked differences in survival between HER2-low and HER2-0 disease after stratification by hormone receptor status [17]. Similarly, a study of early-stage breast cancer found no clear overall survival difference between HER2-low and HER2-0 tumors, although subgroup analyses suggested that the association may vary according to age [18]. Other studies have reported modestly more favorable outcomes among patients with HER2-low tumors, particularly in hormone receptor–positive disease [19]. Differences across studies may reflect variation in hormone receptor distribution, age, disease stage, proliferative activity, treatment exposure, follow-up duration, and analytical methods. Changes in HER2 expression between primary and recurrent tumors may introduce additional heterogeneity when outcomes are evaluated across different disease settings [10]. In our cohort, the more favorable clinicopathological characteristics of HER2-low tumors were not accompanied by significantly different survival outcomes, further illustrating the uncertainty surrounding the independent prognostic contribution of low HER2 expression.

By contrast, advanced clinical stage and hormone receptor status were the covariates most strongly associated with outcome in our multivariable models, whereas the association with high histological grade was attenuated once hormone receptor status was taken into account. This finding underscores the continued importance of established clinicopathological characteristics in breast cancer prognostication. Accordingly, the absence of a statistically significant association for HER2-low status should not be interpreted as definitive evidence that it has no prognostic effect, particularly given the limited number of outcome events and the restricted adjustment set. It should also not be conflated with a lack of clinical relevance, because the prognostic implications of HER2-low expression are conceptually distinct from its value in identifying patients who may benefit from HER2-directed treatment [9,20,21].

From a biological perspective, whether HER2-low breast cancer constitutes a distinct entity remains controversial. Available evidence indicates substantial clinical and molecular heterogeneity within this category, much of which appears to be related to hormone receptor status and intrinsic molecular subtype [6]. HER2-low expression may also change during disease evolution, with transitions between HER2-0 and HER2-low categories occurring between primary and recurrent tumors. Broader discordance in HER2 expression between primary and metastatic disease has also been documented, challenging the interpretation of HER2-low as a stable biological characteristic [10,22].

Diagnostic uncertainty represents an additional concern, particularly when distinguishing IHC 0 from IHC 1+ tumors. This distinction was not originally developed to determine eligibility for HER2-directed therapy, and reproducibility at these low expression levels remains limited. Pathology studies have documented clinically relevant interobserver variability and differences associated with the antibody platform used, emphasizing the importance of standardized interpretation, quality assurance, and specialized training [23,24,25]. In the present study, HER2 classification was based on routine clinical pathology reports without centralized pathological reassessment, which may have introduced additional variability in distinguishing IHC 0 from IHC 1+ tumors. In our cohort, HER2-low tumors accounted for 49.1% of cases without HER2 amplification. Reported proportions using this denominator range from approximately 45% in a series of primary tumors to 60% in metastatic disease [9,10]. This variability is compatible with differences in case mix and with the limited reproducibility of the IHC 0 versus 1+ distinction described above.

Against this background, the current clinical relevance of HER2-low expression appears to be primarily predictive rather than prognostic. Antibody–drug conjugates, particularly trastuzumab deruxtecan, have expanded treatment options for patients with low levels of HER2 expression. DESTINY-Breast04 demonstrated significant improvements in PFS and OS with trastuzumab deruxtecan compared with physician’s choice of chemotherapy in previously treated HER2-low advanced breast cancer [9,26]. These findings supported its incorporation as a treatment option for this population [9,27]. More recent evidence has extended the therapeutic relevance of trastuzumab deruxtecan to hormone receptor–positive metastatic breast cancer with HER2-low or HER2-ultralow expression after endocrine-based therapy [26]. These results illustrate that low HER2 expression may identify tumors that are responsive to a specific therapeutic strategy without necessarily defining a group with a distinct natural history. Our findings are compatible with this distinction, although they cannot exclude a modest independent prognostic effect.

An additional exploratory finding was the higher frequency of CNS metastases in HER2-positive and HER2-negative tumors than in HER2-low disease. The increased occurrence of brain metastases in HER2-positive metastatic breast cancer is well recognized [28]. However, only 11 patients in our cohort had CNS involvement; therefore, the differences observed across HER2 categories should be interpreted cautiously and cannot establish a distinct organ-specific metastatic pattern for HER2-low disease. Available real-world studies and reviews of metastatic HER2-low breast cancer have focused primarily on treatment patterns and clinical outcomes, while evidence defining a consistent metastatic tropism remains limited [29,30].

At the molecular level, HER2-low breast cancer does not appear to represent a uniform intermediate signaling state. Multiomics evidence indicates substantial heterogeneity according to hormone receptor status and intrinsic molecular subtype. In particular, among hormone receptor–negative tumors, basal-like HER2-low disease may resemble HER2-0 breast cancer, whereas selected non-basal-like HER2-low tumors may share molecular features with HER2-positive disease, including PIK3CA mutations and alterations involving PI3K–AKT and ERBB2-related signaling [31]. These observations suggest that the biological behavior of HER2-low tumors may be more strongly influenced by hormone receptor status and intrinsic molecular subtype than by low HER2 expression alone.

This study has several strengths, including its real-world design and comprehensive clinicopathological characterization, which provide clinically relevant evidence from a Latin American population. This regional contribution is important because data describing HER2-low breast cancer among Hispanic and Latin American populations remain limited and are unevenly distributed across countries [12].

However, the single-center nature of the cohort and its location in a high-complexity referral hospital in northeastern Colombia may limit external validity. Patterns of stage at diagnosis, access to pathology testing, availability of systemic therapies, use of HER2-directed treatments, and continuity of oncologic care may differ across regions of Colombia, other Latin American countries, and higher-income healthcare systems. Therefore, the observed distribution of HER2 categories and their association with survival should be extrapolated cautiously to populations with different referral patterns, healthcare access, treatment availability, and clinical characteristics.

Several limitations should be acknowledged. First, the relatively small number of outcome events reduced statistical precision and limited the ability to detect modest differences in survival. The minimum detectable effect analysis indicated that the study could identify only relatively large differences between HER2-low and HER2-negative tumors. Specifically, the minimum detectable hazard ratios were 2.65 for OS and 2.37 for PFS, indicating limited statistical power to detect small-to-moderate prognostic effects. Therefore, the absence of statistically significant associations should not be interpreted as evidence of equivalence or as definitive evidence of no prognostic effect. The limited number of events required a parsimonious multivariable adjustment set that included hormone receptor status but excluded Ki-67 and first-line treatment. Because these characteristics differed across HER2 categories, residual confounding related to proliferative activity and treatment selection cannot be excluded. The adjusted estimates should therefore be interpreted within the limitations of the available covariate set. Detailed treatment-response analyses within the HER2-low subgroup were not performed because this was not the primary objective of the study and several response categories included small numbers of patients, limiting the interpretability of further subgroup comparisons. Future studies with larger and more homogeneous populations should specifically evaluate treatment-response patterns within HER2-low breast cancer.

Additionally, treatment intent and sequence were not systematically captured in the retrospective database, preventing reliable distinction between neoadjuvant, adjuvant, and metastatic systemic therapy. Consequently, treatment-response categories could not be attributed to a specific treatment setting, and the proportions of stage I–III patients receiving neoadjuvant versus adjuvant systemic therapy could not be determined.

The single-center design may limit the generalizability of the findings. Fourth, although PFS was analyzed separately in patients treated with curative intent and in patients with stage IV disease, the time origin remained the date of diagnosis in both strata; the stage I–III estimates therefore correspond conceptually to recurrence-free survival, and the stage IV analysis is limited by its small size and remains exploratory. Follow-up was limited to approximately 40 months, which may be insufficient to capture late recurrences, particularly in hormone receptor–positive breast cancer [32]. This precludes robust assessment of long-term survival. Longer follow-up will be necessary to determine whether prognostic differences between HER2 expression groups emerge over time.

## 5. Conclusions

In this real-world cohort, HER2-low status was not significantly associated with OS or PFS after adjustment for age, clinical stage, histological grade, and hormone receptor status, and the same was true when PFS was analyzed separately in early-stage and metastatic disease. These results come from a Latin American cohort that included patients with disease ranging from early-stage to metastatic, a population for which data are still limited. These findings are consistent with the current view that the main clinical relevance of HER2-low expression may be predictive, through the identification of patients who could benefit from HER2-directed therapies, rather than as an established independent prognostic marker. Nevertheless, the limited number of outcome events and the possibility of residual confounding related to proliferative activity and treatment heterogeneity should be considered when interpreting these findings. Larger studies with longer follow-up and more comprehensive adjustment are needed to clarify the prognostic implications of HER2-low expression.

## Figures and Tables

**Table 1:** Baseline demographic, clinical, and pathological characteristics of breast cancer patients according to HER2 classification.

Variable	HER2-Low (*n* = 143)	HER2-Negative (*n* = 148)	HER2-Positive (*n* = 82)	Total (*n* = 373)	*p* Value
Sex, *n* (%)					0.892
Female	142 (99.3)	147 (99.3)	81 (98.8)	370 (99.2)	
Male	1 (0.7)	1 (0.7)	1 (1.2)	3 (0.8)	
Age (years), median (IQR)	58 (48–67)	53.5 (44.5–64)	54 (47–65)	55 (46–65)	0.110
Health insurance, *n* (%)					0.846
Contributory	35 (24.5)	32 (21.6)	19 (23.2)	86 (23.1)	
Subsidized	108 (75.5)	116 (78.4)	63 (76.8)	287 (76.9)	
Clinical stage, *n* (%)					0.225
I	31 (21.7)	22 (14.9)	7 (8.5)	60 (16.1)	
II	54 (37.8)	63 (42.6)	35 (42.7)	152 (40.8)	
III	44 (30.8)	52 (35.1)	33 (40.2)	129 (34.6)	
IV	14 (9.8)	11 (7.4)	7 (8.5)	32 (8.6)	
Histological grade, *n* (%)					
1	15 (10.5)	13 (8.8)	2 (2.4)	30 (8.0)	<0.001
2	99 (69.2)	72 (48.6)	45 (54.9)	216 (57.9)	
3	29 (20.3)	59 (39.9)	35 (42.7)	123 (33.0)	
Not reported	0 (0.0)	4 (2.7)	0 (0.0)	4 (1.1)	
ER, median (IQR)	1.0 (0.9–1.0)	0.95 (0.65–1.0)	0.9 (0.2–1.0)	0.98 (0.8–1.0)	<0.001
PR, median (IQR)	0.8 (0.4–0.9)	0.8 (0.30–0.9)	0.4 (0.1–0.7)	0.7 (0.3–0.9)	<0.001
Ki-67, median (IQR)	0.2 (0.2–0.5)	0.3 (0.15–0.8)	0.4 (0.3–0.6)	0.3 (0.2–0.7)	<0.001
Hormone receptor status, *n* (%)					0.001
HR-positive	124 (86.7)	116 (78.4)	53 (64.6)	293 (78.6)	
HR-negative	19 (13.3)	32 (21.6)	29 (35.4)	80 (21.4)	
Response to first-line treatment, *n* (%)					0.076
Complete response	44 (30.8)	50 (33.8)	35 (42.7)	129 (34.6)	
Partial response	71 (49.7)	70 (47.3)	24 (29.3)	165 (44.2)	
Stable disease	5 (3.5)	4 (2.7)	1 (1.2)	10 (2.7)	
Progressive disease	5 (3.5)	8 (5.4)	4 (4.9)	17 (4.6)	
Not evaluated	18 (12.6)	16 (10.8)	18 (22.0)	52 (13.9)	
Distant metastasis, *n* (%)					0.388
No	125 (87.4)	123 (83.1)	73 (89.0)	321 (86.1)	
Yes	18 (12.6)	25 (16.9)	9 (11.0)	52 (13.9)	
Number of metastases, *n* (%)					0.064
1	6 (4.2)	15 (10.1)	1 (1.2)	22 (5.9)	
2	5 (3.5)	3 (2.0)	2 (2.4)	10 (2.7)	
3	1 (0.7)	3 (2.0)	2 (2.4)	6 (1.6)	
4	3 (2.1)	1 (0.7)	1 (1.2)	5 (1.3)	
5	0 (0.0)	1 (0.7)	3 (3.7)	4 (1.1)	
7	2 (1.4)	0 (0.0)	0 (0.0)	2 (0.5)	
10	1 (0.7)	2 (1.4)	0 (0.0)	3 (0.8)	
N/A	125 (87.4)	123 (83.1)	73 (89.0)	321 (86.1)	

Values are presented as *n* (%) or median (interquartile range, IQR). *p*-values were calculated using the chi-square test or Fisher’s exact test for categorical variables and the Kruskal–Wallis test for continuous variables. HER2 classification includes HER2-low, HER2-negative, and HER2-positive groups. HER2, human epidermal growth factor receptor 2; ER, estrogen receptor; PR, progesterone receptor; Ki-67, proliferation index, N/A, not applicable.

**Table 2:** Distribution of metastatic sites according to HER2 classification in patients with breast cancer.

Metastatic Site	HER2-Low (*n* = 18)	HER2-Negative (*n* = 25)	HER2-Positive (*n* = 9)	Total (*n* = 52)	*p* Value
Bone	12 (66.7)	9 (36.0)	6 (66.7)	27 (51.9)	0.087
Lung	10 (55.6)	8 (32.0)	6 (66.7)	24 (46.2)	0.124
Liver	3 (16.7)	6 (24.0)	2 (22.2)	11 (21.2)	0.842
Brain/CNS	1 (5.6)	5 (20.0)	5 (55.6)	11 (21.2)	0.011
Lymph nodes	3 (16.7)	5 (20.0)	2 (22.2)	10 (19.2)	0.934
Pleura	3 (16.7)	1 (4.0)	1 (11.1)	5 (9.6)	0.375
Other sites	0 (0.0)	3 (12.0)	0 (0.0)	3 (5.8)	0.179

Values are presented as *n* (%). Percentages in each HER2 column were calculated using the number of patients with metastatic disease within that HER2 group as the denominator: HER2-low, *n* = 18; HER2-negative, *n* = 25; and HER2-positive, *n* = 9. Percentages in the Total column were calculated using all patients with metastatic disease (*n* = 52) as the denominator. Patients could have more than one metastatic site; therefore, percentages may sum to more than 100%. Lymph nodes refer to distant lymph-node involvement and do not include regional lymph-node involvement used for TNM staging. *p*-values were calculated using the Chi-square test or Fisher’s exact test, as appropriate. CNS: central nervous system.

**Table 3:** First-line treatment modalities according to HER2 classification.

Treatment Modality	HER2-Low (*n* = 143)	HER2-Negative (*n* = 148)	HER2-Positive (*n* = 82)	Total (*n* = 373)	*p* Value
Anthracyclines (AC ± variants)	23 (16.1)	20 (13.5)	6 (7.3)	49 (13.1)	<0.001
Anthracycline dose-dense (AC/dd)	34 (23.8)	40 (27.0)	12 (14.6)	86 (23.1)	
Carboplatin + taxane (Carbotaxol ± variants)	9 (6.3)	6 (4.1)	0 (0.0)	15 (4.0)	
Platinum-based (other)	1 (0.7)	1 (0.7)	0 (0.0)	2 (0.5)	
Immunotherapy regimen (KEYNOTE 522 ± variants)	2 (1.4)	4 (2.7)	0 (0.0)	6 (1.6)	
Anti-HER2 regimens (TCHP/THP/CLEOPATRA/dual blockade)	3 (2.1)	1 (0.7)	51 (62.2)	55 (14.7)	
Hormonal therapy (±CDK4/6 inhibitors)	12 (8.4)	15 (10.1)	0 (0.0)	27 (7.2)	
Surgical management only	21 (14.7)	40 (27.0)	5 (6.1)	66 (17.7)	
Other treatments	36 (25.2)	13 (8.8)	3 (3.7)	52 (13.9)	
Not reported	2 (1.4)	8 (5.4)	5 (6.1)	15 (4.0)	

Values are presented as *n* (%), with percentages corresponding to column totals. Treatment modalities were grouped according to clinical management strategies. *p*-values were calculated using the Chi-square test or Fisher’s exact test, as appropriate. AC: anthracyclines; AC/dd: dose-dense anthracyclines; TCHP: docetaxel, carboplatin, trastuzumab, and pertuzumab; THP: taxane, trastuzumab, and pertuzumab; CDK4/6: cyclin-dependent kinase 4/6 inhibitors.

**Table 4:** Multivariable Cox regression analysis for overall survival and progression-free survival.

Variable	OS HR (95% CI)	*p* Value	PFS HR (95% CI)	*p* Value
HER2 classification (ref Negative)				
HER2-low	0.67 (0.31–1.45)	0.312	0.77 (0.40–1.49)	0.443
HER2-positive	0.66 (0.30–1.49)	0.320	0.73 (0.36–1.49)	0.382
Age (years)	1.03 (1.00–1.05)	0.018	1.01 (0.99–1.03)	0.276
Clinical stage III–IV (ref I–II)	6.09 (2.64–14.02)	<0.001	4.24 (2.21–8.10)	<0.001
Histological grade 3 (ref 1–2)	2.04 (0.96–4.35)	0.065	1.71 (0.90–3.28)	0.102
Hormone receptor–positive (ref negative)	0.43 (0.21–0.88)	0.020	0.57 (0.30–1.06)	0.078

Values are presented as hazard ratios (HRs) with 95% confidence intervals (CIs). The OS model included 368 patients and 41 deaths, whereas the PFS model included 368 patients and 53 events. Multivariable analyses were based on complete cases for the covariates included in each model and were adjusted for age at diagnosis, clinical stage (III–IV vs. I–II), histological grade (grade 3 vs. grades 1–2), hormone receptor status (positive vs. negative), and HER2 classification. HER2-negative was used as the reference category.

## Data Availability

The datasets generated and analyzed during the current study are not publicly available due to institutional privacy policies and patient confidentiality but are available from the corresponding author upon reasonable request.

## Abbreviations

AC	Anthracycline and cyclophosphamide
AC/dd	Dose-dense anthracycline and cyclophosphamide
ADC	Antibody–drug conjugate
BC	Breast cancer
CDK4/6	Cyclin-dependent kinases 4 and 6
CI	Confidence interval
CNS	Central nervous system
ER	Estrogen receptor
FCV	Fundación Cardiovascular de Colombia
HER2	Human epidermal growth factor receptor 2
HIC	Hospital Internacional de Colombia
HR	Hazard ratio
IHC	Immunohistochemistry
IQR	Interquartile range
ISH	In situ hybridization
N/A	Not applicable
OS	Overall survival
PFS	Progression-free survival
PR	Progesterone receptor
REDCap	Research Electronic Data Capture
TCHP	Docetaxel, carboplatin, trastuzumab, and pertuzumab
THP	Taxane, trastuzumab, and pertuzumab
TNM	Tumor, node, metastasis
